# Testing Mars-inspired operational strategies for semi-autonomous rovers on the Moon: The GeoHeuristic Operational Strategies Test in New Mexico

**DOI:** 10.1555/mars.2011.0002

**Published:** 2011-12-29

**Authors:** R. Aileen Yingst, B. A. Cohen, L. Crumpler, M. E. Schmidt, C. M. Schrader

**Affiliations:** 1 Planetary Science Institute, Tucson, AZ, 85719, USA; 2 Space Science Office, Marshall Space Flight Center, Huntsville, AL, 35812, USA; 3 New Mexico Museum of Natural History and Science, Albuquerque, NM, 87104, USA; 4 Dept. of Earth Sciences, Brock University, St. Catharines, ON, L2S 3A1, Canada

## Abstract

**Background:**

We tested the science operational strategy used for the Mars Exploration Rover (MER) mission on Mars to determine its suitability for conducting remote geology on the Moon by conducting a field test at Cerro de Santa Clara, New Mexico. This region contains volcanic and sedimentary products from a variety of provenances, mimicking the variety that might be found at a lunar site such as South Pole-Aitken Basin.

**Method:**

At each site a Science Team broke down observational “days” into a sequence of observations of features and targets of interest. The number, timing, and sequence of observations was chosen to mimic those used by the MERs when traversing. Images simulating high-resolution stereo and hand lens-scale images were taken using a professional SLR digital camera; multispectral and XRD data were acquired from samples to mimic the availability of geochemical data. A separate Tiger Team followed the Science Team and examined each site using traditional terrestrial field methods, facilitating comparison between what was revealed by human versus rover-inspired methods.

**Lessons Learned:**

We conclude from this field test that MER-inspired methodology is not conducive to utilizing all acquired data in a timely manner for the case of any lunar architecture that involves the acquisition of rover data in near real-time. We additionally conclude that a methodology similar to that used for MER can be adapted for use on the Moon if mission goals are focused on reconnaissance. If the goal is to locate and identify a specific feature or material, such as water ice, a different methodology will likely be needed.

## Introduction

### Background

Robotic semi-autonomous roving vehicles are designed to remotely perform many of the functions of a field geologist, and as such, they are crucial tools in the geologic exploration of extraterrestrial surfaces. The operational strategies used in remote, rover-driven field studies are derived from terrestrial field methods (we here define operational strategy as the manner and sequence in which instruments or other tools are used to answer scientific questions). These methods must necessarily be adjusted for the unique problems associated with conducting work remotely, and for the abilities of the rovers in their specific environments. For example, the operational strategies currently in use for the Mars Exploration Rover (MER) mission were designed and refined in part through a series of field tests conducted on Earth prior to landed operations (Squyres et al. 2003), and in part through rover simulations (*e.g.*, Stoker 1998; Stoker et al. 2001; Thomas et al. 2001; Arvidson et al. 2002; Jolliff et al. 2002; Stoker et al. 2002). The resulting set of strategies has provided the framework for many MER operational decisions. However, scientists have adapted the protocol in response to unexpected circumstances subsequent to landing, and have developed more efficient ways to utilize the instrument package as well as the individual instruments. The evolution of MER operational strategies over the mission demonstrates the importance of evaluating and modifying methods used in semi-autonomous rover-driven fieldwork, to maximize science return.

### MER operational strategies as a potential model for lunar rover activities

The Mars Exploration Rover mission represents the most extensive body of experience conducting field geology remotely on another terrestrial body with a semi-autonomous rover. As such, the operational strategies used for MER on Mars can be assessed to understand best practices for conducting remote geology on Mars and other terrestrial bodies such as the Moon, provided differences in environment, mission architecture and science goals are taken into account. Differences specific to the Moon include the nature of the terrain, the physical properties of the regolith, the diurnal cycle and wider temperature variation in general, and the lack of atmosphere. Technical issues to consider include the shorter communications time between Earth and the Moon, the question of how that communication will occur (direct-to-Earth, relay to an orbiter, or some other method), the problems inherent in the temperature variation over a tidally-locked airless body, and issues unique to landing site location (a farside site would require a different communications system, for example, than a nearside location). Mission architectures that have been proposed include utilizing rovers as “scouts” to reconnoiter a site prior to human landing, as tools used concurrently with and by on-site humans, as systematic surveyors subsequent to human activity, and as stand-alone reconnaissance craft (like the MER model) (*e.g.*, Bualat et al. 2006; Fong et al. 2008; Deans et al. 2009). Each of these possibilities may require a different outlook and strategy for conducting science operations.

Finally, the driving science questions for the Moon posed by sources such as the Decadal Survey and other works (Jolliff et al. 2000; Exploration Systems Architecture Study 2005; National Research Council 2007; Drake et al. 2007) are vastly different from those that informed the MER science goals. Most proposed and actual semi-autonomous rover activities for Mars have centered around gathering data that would illuminate the overarching issue of that planet’s present and past suitability for life (*e.g.*, Squyres et al. 2003). The Moon, by contrast, is a small, airless, rocky body that retains a record of the early history of impacts in the solar system, as well as a record of differentiation and a primary crust. The Moon is thus a geologic end-member whose geology can reveal clues regarding planetary differentiation, basaltic volcanism in its earliest stages, and impact activity in the inner solar system. Also important in the exploration of the Moon is the identification of potential resources, most notably water ice and volcanic products.

For the Geo-Heuristic Operational Strategies Test (GHOST), we tested current MER semi-autonomous rover operations strategies in terms of their usefulness, efficiency and response when executed within defined parameters that mimic aspects of a lunar environment, including rapid communication and variety and provenance of geologic materials. The primary objective of the GHOST experiment was to determine whether MER-derived field methodologies utilized in a lunar analog environment provided sufficient data to identify, characterize and interpret lunar analog geologic materials that revealed the field site’s stratigraphy and geologic history.

## Approach

Lunar analog field tests building on lessons learned from Lunakhod (*e.g.*, Cadogan 1981; Bourke et al. 1996; Ulrich et al. 1996), have included developing methodologies (Greeley et al. 1994; Stoker and Hine 1996), mobile simulations of geologic fieldwork with a Lunakhod-class rover in a lunar analog environment (Taylor et al. 1995; Whittaker et al. 1997; Stoker 1998), testing potential instrumentation and robotics (Whittaker et al. 1997; Roman et al. 2004; Glass et al. 2006; Lee et al. 2007 and references therein), assessing operator performance and science return under teleoperation conditions (Kunii et al. 2001; Miller and Machulis 2005) and studying the interaction of a science team during simulated operations (Thomas et al. 2001; Lofgren et al. 2009). More recent activities have included testing specific mission architectures such as various permutations of rover-assisted human surface activities (*e.g.*, Bualat et al. 2006; Fong et al. 2008; Deans et al. 2009). For the bulk of these tests, the common strategy has been to use a rover mock-up armed with a suite of instruments, with an engineering team in the field (sometimes including humans performing as “astronauts”) and a “blind” team off-site conducting or supporting the science.

By contrast, in this study we assessed the performance of science-driven operational strategies alone, rather than any specific instruments or rover body, in facilitating and optimizing science return. Commercial, off-the-shelf instruments that provided similar information to that produced by flight-ready instruments were taken to a terrestrial analog field site and used to acquire data; humans were utilized for mobility. The manner and sequence in which those instruments were used to reconnoiter a geologic site, choose targets of interest and investigate specific geologic questions regarding those targets, were modeled after those currently utilized for MER on Mars. However, the geologic site was chosen so that selected variables of a lunar environment could be introduced, and these variables were chosen based on science alone. That is, the site was considered an acceptable lunar analog if it contained the type, variety and provenance of geologic materials expected from our lunar site. Results regarding whether and how these operational strategies were suitable for lunar rover activities were thus independent of technical issues that might have been introduced by equipment failure, problems with traversibility or environmental concerns such as weather. Finally, this approach models traditional terrestrial geologic fieldwork in that it is heuristic; that is, problem-solving takes place through experimentation, knowledge is gained by experience.

## Methodology

### Instruments

As currently conducted, MER rover science activities are organized by operational activities conducted over a certain number of Martian days (referred to as sols). The suite of instruments available for these activities serves functions likely similar to those that will be required for many lunar rover missions. These functions include: (1) imaging capabilities for hazard avoidance and navigation; (2) multispectral imaging capabilities for understanding geomorphology and mineralogy; and (3) instruments for geochemical analysis of major and minor elements. MER instruments available for science activities thus include the 2.2 mrad/pixel angular resolution, 180° field-of-view hazard-avoidance cameras (Hazcams); the 0.77 mrad/pixel angular resolution, 45×45 degree field-of-view navigational cameras (Navcams) (Maki et al. 2003); the 13-filter, UV-Vis stereo panoramic camera (Pancam (Bell et al. 2003)), the 31 μm/pixel resolution, monocolor Microscopic Imager (MI (Herkenhoff et al. 2003)); Miniature Thermal Emission Spectrometer (MTES) (Christensen et al. 2003), designed to provide mineralogical and thermophysical data on surface materials; and the alpha particle X-ray and Mössbauer spectrometers for determining major and minor elemental composition (APXS and MB) (Klingelhöfer et al. 2003; Rieder et al. 2003). Detailed specifications of these instruments are enumerated in the references noted. We required an imager capable of acquiring images at or better than the resolution of the MER imagers. Pancam provides images at a range of spatial resolutions from sub-mm to cm-scale resolution; as an example, an image taken at a 70° downlook angle at a distance of 1.5 m from the surface would have a resolution of ~0.4 mm/pixel (Bell et al. 2003)). The MI is a fixed-focus imager providing best resolution at ~31 μm/pixel (Herkenhoff et al. 2003). To cover this range of resolutions, we chose a professional single lens reflex (SLR) digital camera with interchangeable lens capability and megapixel imaging, including a 100 mm f/2.8 macro lens. The focal range of the camera and the selection of lenses allowed us to image features at all scales used by the MER imagers. A working distance from the front of the lens to the target of ~15 cm allowed 24 × 36 mm images at ~10 μm/pixel to be taken using ambient light; this resolution is within the range of the MI and the MArs HandLens Imager (MAHLI) for the Mars Science Laboratory rover science package (Edgett et al. 2005). Pancam can acquire mineralogical data through the use of 13 geologic filters that span the spectral range from 400 to 1100 nm. Geochemical data from the MER landing sites is acquired through the APXS and MB instruments. The APXS uses an X-ray detector to measure the presence and concentration of elements from sodium up to yttrium, and backscattered alpha spectra to acquire data on carbon and oxygen. The MB irradiates surface materials and measures backscatter radiation to detect and measure the concentration of iron-bearing phases and magnetic particles. To mimic the multispectral and geochemical analysis capabilities of these MER instruments, we utilized an ALTA II field UV-Vis reflectance spectrometer and a Terra field-portable XRD. The ALTA II is a multiband photometer that measures reflectances in separate, non-overlapping ranges of wavelength centered on 470, 525, 560, 585, 635, 660, 700, 735, 810, 880 and 940 nm. These peaks are sensitive to many Fe-bearing species. The Terra field-portable XRD has a 1024 × 256, 2-D peltier-cooled CCD camera. This field instrument is fully self-contained and provides reasonably accurate identification of major, minor and trace components with minimal sample preparation. This instrument acquires data that is generally similar to CheMin on the Mars Science Laboratory rover.

### Observational activities

Common MER observation campaigns have been classified into two categories: (1) a “traverse mode” typical of operations performed when the goal is to drive to a distant location but the rover may stop for a limited time (3–5 sols) to examine targets of opportunity along the way; and (2) a 25–40 sol “survey mode” that has been practiced when examining a larger feature of high scientific value (Yingst et al. 2008). In such a case, the time spent examining the site has been constrained by the limitations of the environment, rather than by more pressing science priorities. An example of the former mode includes Spirit’s traverse up the Columbia Hills (*e.g.*, Arvidson et al. 2006), a period when many sites were left before it was optimal to do so (a situation that may mirror science during teleoperation (Taylor et al. 1995; Stoker 1998)). Examples of the latter mode include the investigations of Home Plate by Spirit prior to the last martian winter (Arvidson et al. 2007) and the detailed examination of the Burns formation at the Opportunity landing site (Squyres et al. 2005; Grotzinger et al. 2005), which included ~35 targets in 8 locations.

#### MER traverse mode observations

**Figure 1:**
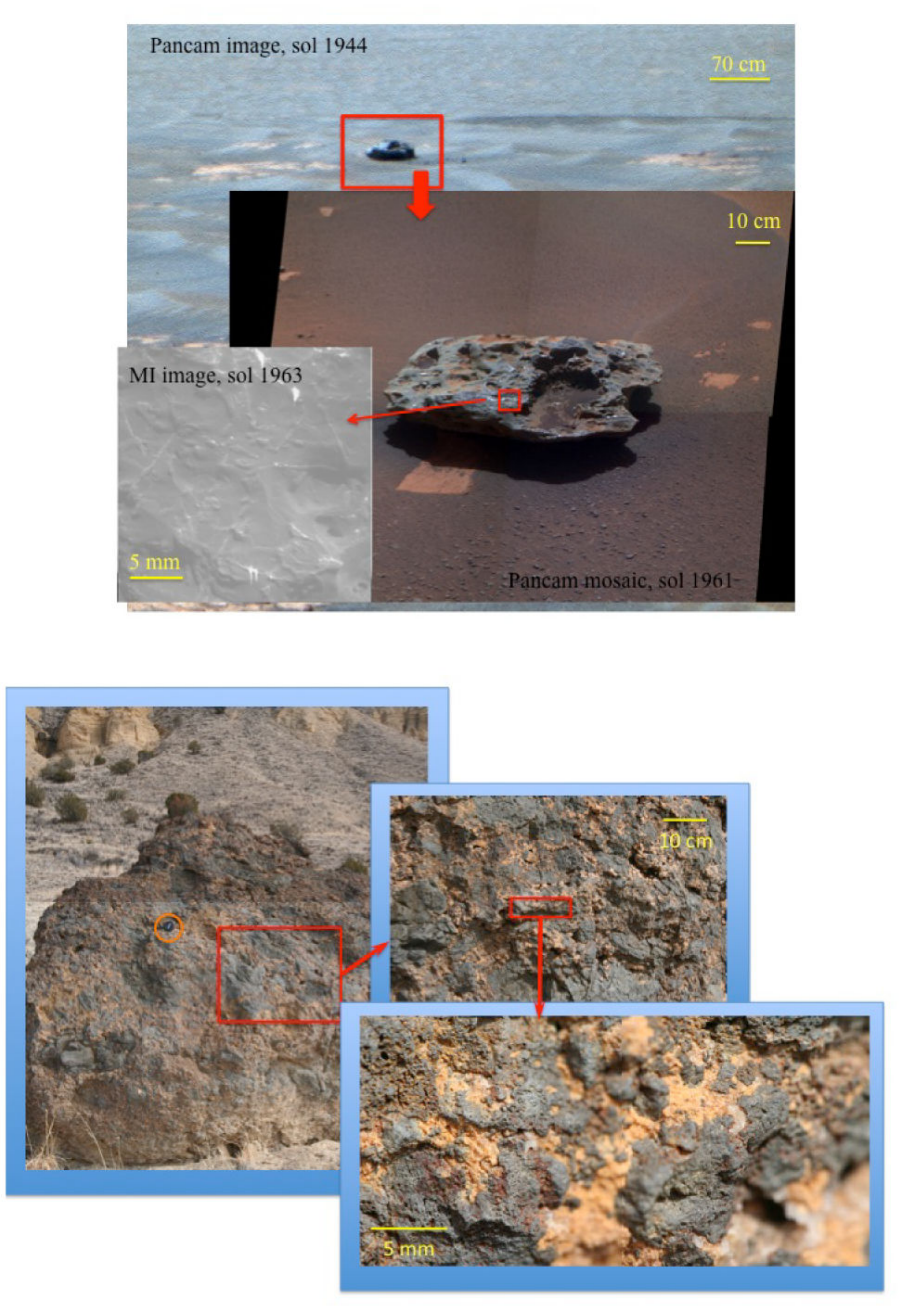
Set of images at nested resolutions taken by (a) the MER rover Opportunity; and (b) the GHOST Science Team. (a) is a sequence of images of the meteorite Block Island taken by Pancam and the Microscopic Imager between sols 1944–1961. (b) is a sequence of images acquired at Station 2, the welded-cinder boulder Chorizo. Circled in the coarsest resolution image (left) is a lenscap for scale.

When MER is in traverse mode, a systematic set of observations is conducted before and after every drive. When a target of interest is found, observation begins with approach-imaging at outcrop and feature resolution, supported by prior panoramic imaging (observations acquired by Pancam and the Navcams, and less often by the Hazcams). Due to data rate, data volume and power constraints, some, but not all, of these images may be taken using three Pancam multispectral filters to mimic color; more rarely, an image is acquired using the entire range of multispectral filters. Three to five targets of opportunity within an area of about 1 m^2^ may be selected based on data observed at this resolution. These targets are then examined by the suite of instruments mounted on the rover arm; they are imaged by the MI and analyzed geochemically by the APXS and MB. Figure 1 shows an example of this range of image resolutions for targets at Mars (a) and for the GHOST field test (b).

#### MER survey mode observations

Survey mode also begins with approach-imaging at outcrop resolution, supported by previous panoramic lower-resolution images. The number and resolution of images is greater, however, and targeted to contextualize individual units or features in the larger feature of interest. One or more targets in each unit or feature are also imaged at high spectral and spatial resolution before they are examined in detail by geochemical and handlens-resolution instruments.

#### MER strategies adapted for the GHOST field test

**Figure 2:**
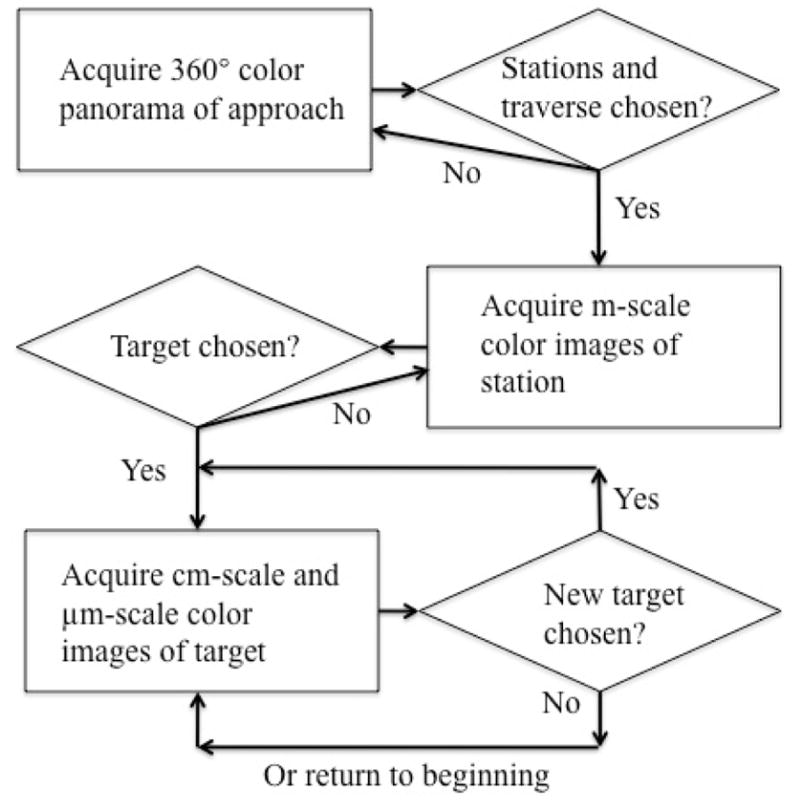
Generalized data acquisition strategy for the Science Team. Rectangles represent data acquisition points, while diamonds represent decisional points. The decisional path is based on Squyres et al. (2003), and adapted from MER operational strategies as executed during the landed mission.

The traverse mode has been the mode utilized most frequently during the MER mission, and was thus adapted for use in this field experiment (the survey mode will be tested in future work). The following strategy was adopted: (1) acquire 360° of panoramic pre-approach images and from these, choose up to ten sites that lie within a reasonable “traverse” area, for closer examination; (2) acquire m-scale images of sites and from these choose three targets that potentially best represent materials of interest; (3) acquire images of selected targets at cm-scale resolution and then at 10 μm/pixel; (4) sample targets and analyze for composition. Steps 2–4 were followed for each site selected in step 1. Figure 2 shows this data acquisition path. Additionally, because of the large effect it would potentially have on science operations, we chose to conduct MER-derived operational strategies in near real-time communications, mimicking a likely lunar mission scenario. For this experiment, the delay between commanding the “rover” and receiving requested data was typically 5 minutes and no more than 15 minutes.

## Field site

### Choice of field site

There are numerous sites that have been previously studied as field analogs for a variety of lunar environments, including desert areas (Whittaker et al. 1997; Miller and Machulis 2005), Haughton Crater (*e.g.*, Glass et al. 2006; Lee et al. 2007; Fong et al. 2007, Meteor Crater (Diftler et al. 2007) and numerous locations in Hawaii (Cruikshank and Wood 1972; Taylor et al. 1995; Stoker 1998). For this field test, we assumed South Pole-Aitken Basin as the potential lunar environment, as this is a site on the Moon that is considered a high-priority target for semi-autonomous rover activities in the near future (Exploration Systems Architecture Study 2005; National Research Council 2007), and one that serves as a scientific and operational bounding case. SPA is the largest recognizable impact structure in the solar system, as well as being the oldest (Wilhelms 1987) and deepest (Spudis et al. 1994; Wieczorek and Phillips 1999) basin on the Moon. The basin floor, likely representing a melt sheet, is anomalously mafic (Jolliff et al. 2000; Petro and Pieters 2004). The mineralogy of the basin floor is currently interpreted to be noritic (lower crust) (Pieters et al. 1997, 2001), making it a natural site of interest for addressing several fundamental questions related to the composition and vertical stratigraphy of the lunar lower crust and megaregolith. SPA is also a unique location to study possible variations in the age, composition and extent of volcanic eruptive events on the Moon, as the discrete mare deposits therein show potential variations in composition (Spudis et al. 1984; Gaddis et al. 1995; Yingst et al. 1998, 1999, 2001; Gillis and Spudis 2000), lateral and vertical mixing relationships (Li and Mustard 2003, 2005), age and morphology (Whitford-Stark 1979, 1982; Gaddis 1981; Hawke et al. 1991; Head and Wilson 1992; Yingst et al. 1997, 1998), and pyroclastic activity (Head et al. 2000; Pieters et al. 2001). In terms of applied science, pyroclastics, along with mare basalt deposits, are potential sources of the important lunar resources iron and titanium, and are an enriched source of volatile elements such as Ag, Cd, Zn and Br, elements that are relatively rare on the rest of the Moon (Baedeker et al. 1974; Wasson et al. 1976; Delano 1986; Duke et al. 2006). Thus, a terrestrial site analogous to SPA for this study would include the existence and accessibility of: (1) resource-rich pyroclastics; (2) basaltic volcanic deposits with a variety of morphologies, ages and minerals to test the level at which a rover could identify such differences; and (3) non-volcanic rocks dissimilar to overlying deposits. Cerro de Santa Clara, a volcanic plug in the Rio Puerco valley of New Mexico, fits all of these criteria.

### Geologic setting of field site

Cerro de Santa Clara, in the Rio Puerco Valley west of Albuquerque, New Mexico, is one of several Rio Puerco volcanic plugs or necks that erupted during the late Cenozoic in association with the Mount Taylor volcanic field (Crumpler 1982; Crumpler et al. 2007; Hallett et al. 1997). Underlying the Cenozoic volcanoes are the Cretaceous Mancos Shale and basal Point Lookout Sandstone of the Mesaverde Group. These are a transgressive sequence (Lucas et al. 1992) deposited during emergence of an epicontinental marine basin. Cerro de Santa Clara is an elongate volcanic neck 400 m × 100 m in map plan and 120 m from the base to the summit, that appears to have developed along a former north-northeast-trending dike. The plug consists of non-vesicular, fine- to medium-grained basalt in massive section and capping a local sequence of Mesaverde shales and lenticular sandstones. Lapilli tuffs, agglomerates, bombs, and vent breccias are all present. Exposure of the contact with the underlying Mesaverde units on the south end show that the initial vent activity consisted of some basaltic ash and associated phreatomagmatically comminuted basaltic lithics and sandstone occurring as a greenish-colored outcrop. Immediately overlying this zone (less than 40 m wide) and cutting through it as a dike locally are alkali basaltic intrusions and local extrusions flaring upward to form the predominant cap rock of the elongate plug. Around the periphery, mostly in the debris shed off the very top are welded scoria deposits locally intermixed with palagonitized sandy to fine clay matrix materials.

**Figure 3:**
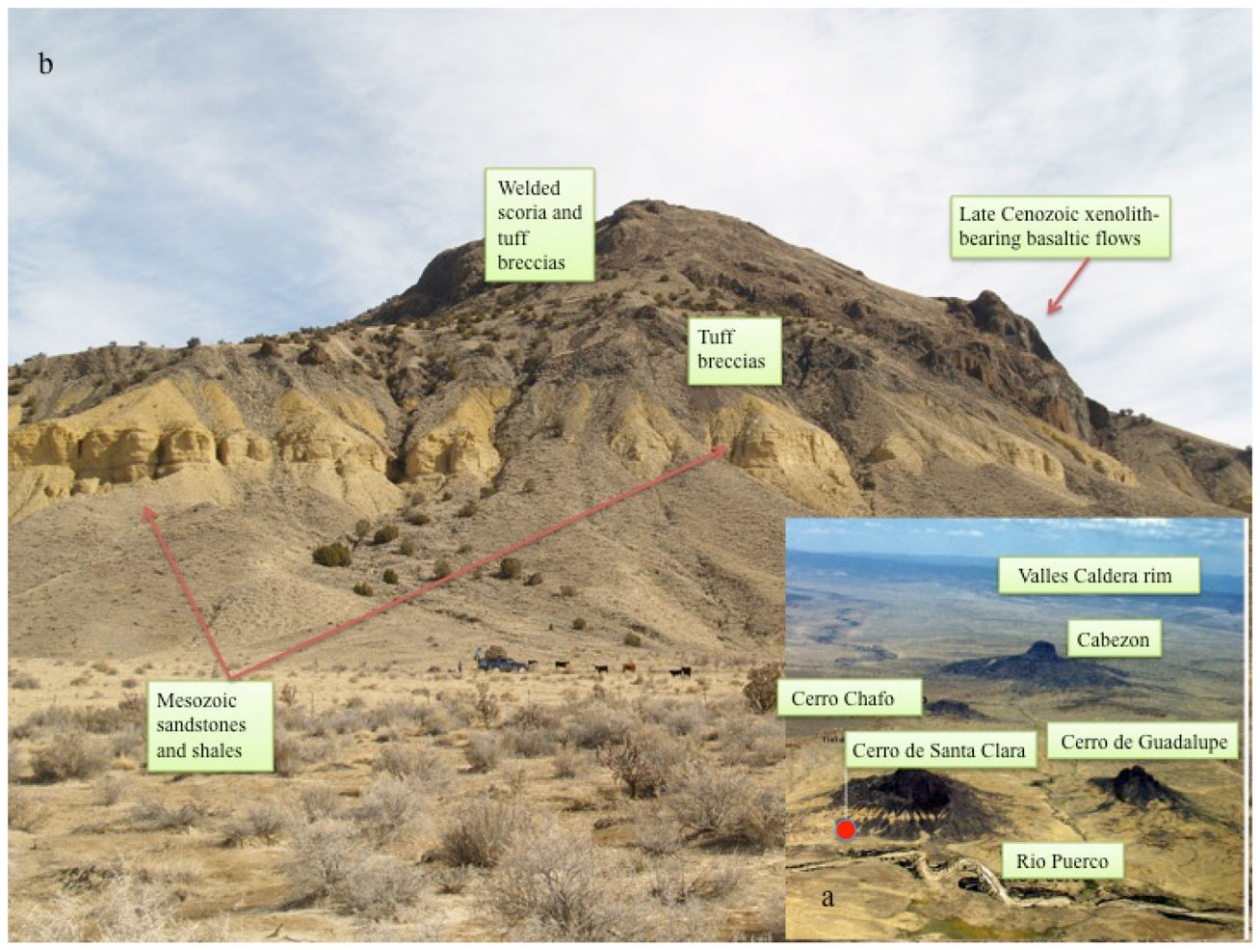
Aerial view (a) and panoramic image (b) of the study site, showing primary geologic units. Red circle in (a) shows the location of Base Camp.

Most of Rio Puerco necks contain one or several varieties of mantle-derived, ultramafic xenoliths and xenocrysts (Brown 1969; Kudo et al. 1972; Wilshire et al. 1988; Porreca et al. 2006), a few uncommon crustal xenoliths, mostly of felsic granulite and granite (Hallett 1992), and in those bearing phreatomagmatic lithologies, xenoliths of shallow Mesozoic rocks. The latter are mostly Dakota and Mesaverde sandstones and shales in various states of thermal alteration. The mantle xenoliths include peridotites, websterites, dunites, harzburgites, clinopyroxenites and orthopyroxenites. Red spinel is a common accessory phase in the peridotites. Few to no reaction rims are present between the nodules and the enclosing basalt. Xenoliths enclosed by basalt are common, but they also occur as separate clasts in the debris shed from plugs. Figure 3 shows the broad range of geologic materials at this site, including massive and pyroclastic basalts, sandstones and shales, and xenoliths with a variety of mineralogies.

## Structure and performance of the experiment

### Team organization and observational structure

Two teams conducted fieldwork during this experiment. The Science Team (two scientists and a field assistant) simulated a scenario in which rover activities are run by an off-site science “backroom” (this simulation would also be generally applicable to one in which astronauts within a habitat controlled rover activities). The team conducted a set of planned measurements (outlined in Table 1) based on common MER observational sequences. Two additional geologists comprised a Tiger Team. The objective of this team was to follow the same traverse and analyze the same targets as the Science Team, but using the traditional terrestrial field methods upon which rover operations were originally based (*e.g.*, walking reconnaissance, handlens observation and analysis, sample collection using a rock hammer). The Tiger Team was allowed to make additional observations based on information gathered studying the original targets as long as time permitted. By comparison, the Science Team, though in the field, had access only to the images acquired by the “rover” and was required to make decisions for follow-on observations based on that data alone. To avoid as much as possible any bias introduced due to areas of expertise, the members of each team were changed around between test days.

The experiment comprised two days of fieldwork (a dry run to a different location to identify and solve any logistical issues, and the actual field test) and one day of debriefing for both teams. Prior to the field experiment, “orbital” photos of the region derived from Google Earth were provided to all team members to familiarize them with the regional geologic context. A local team member scouted specific field locations, so that only one team member would be familiar with the site; this team member was not a part of either the Science Team or the Tiger Team, but provided in-depth knowledge of the site during the debrief.

### Science Team observations

**Figure 4:**
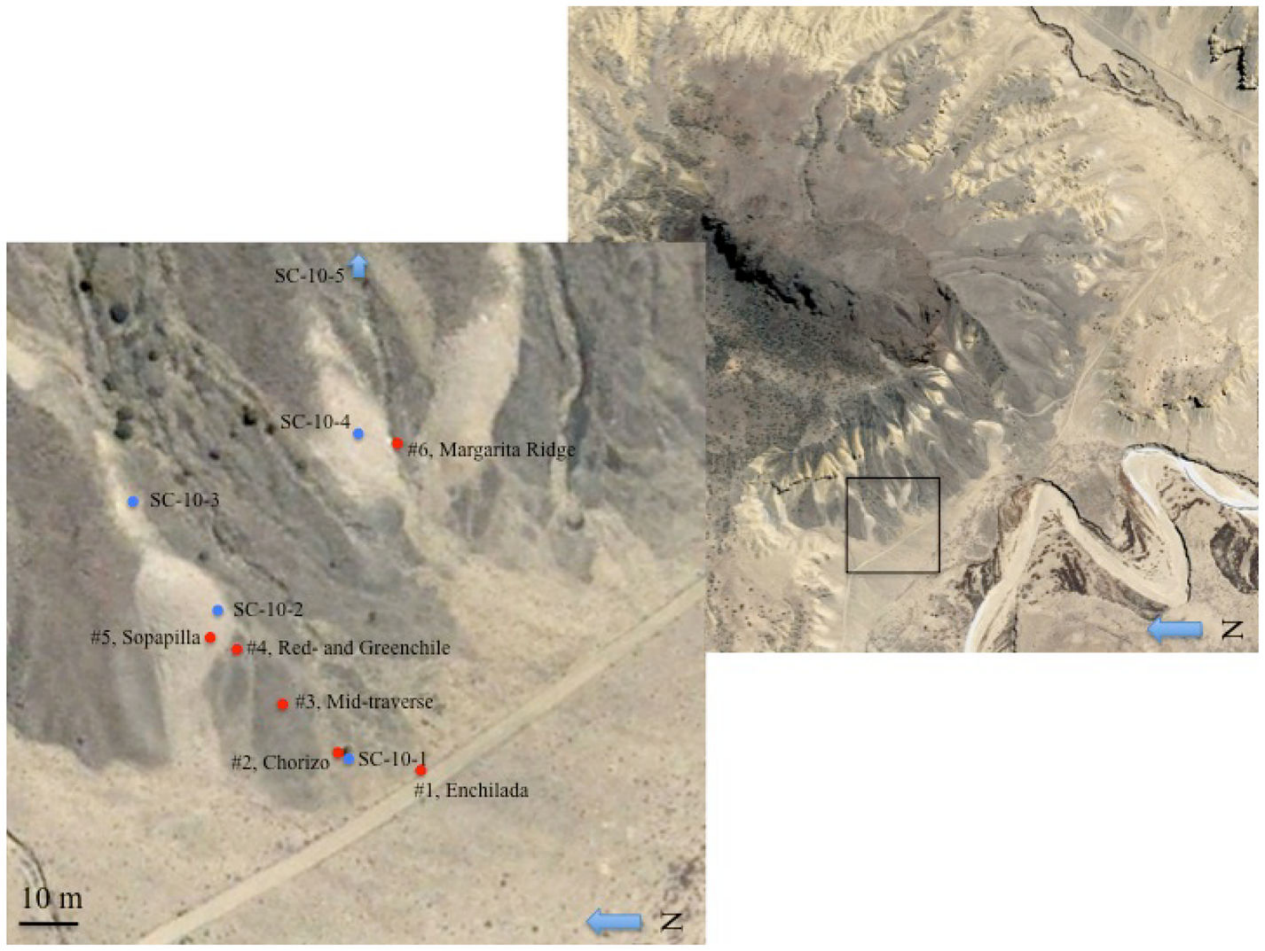
Traverse path and observational stations for the Science Team (shown in red); and the Tiger Team (shown in blue). Station names correspond to those listed in the text; stations were visited in numerical order. Note that because the traverse of the Tiger Team encompasses that of the Science Team, but does not duplicate it, some stations have two designations. The boxed area in the right image is the region shown in the left image.

**Figure 5:**
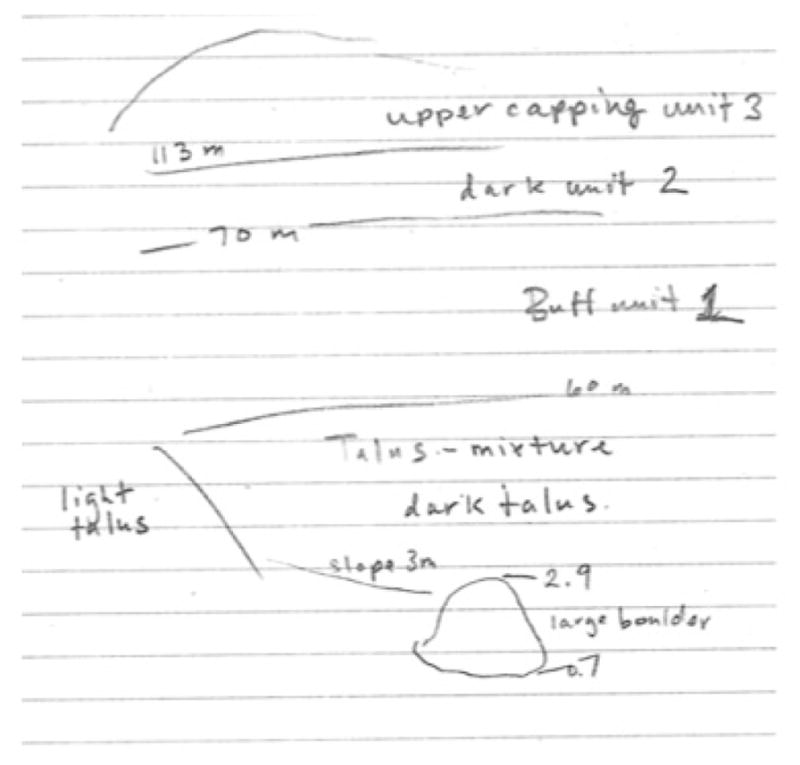
Stratigraphy of the site as hypothesized by the Science Team.

Both teams were transported to the field site together. The Science Team set up a Base Camp from which to make observational decisions, and immediately acquired a 360° panorama of the site and elevation data (assumed to be available from rover navigational and orbital data). From this, the team sketched a first-order stratigraphic section and selected a preliminary traverse, choosing six observation “stations” that the team believed sampled the diversity of stratigraphic and volcanic units; the field assistant “rover” then traversed in order to each “station”. The traverse is shown in Figure 4, while the stratigraphic sketch (showing a strong correlation to the units labeled in Figure 3) is shown in Figure 5. The Science Team provided this preliminary traverse path and set of stations to the Tiger Team. The Tiger Team was aware of the Science Team’s traverse plan, but chose to make its own traverse, which did not match it.

The Science Team took detailed observations of materials at each of the six stations and made interpretations; these are enumerated in Table 1. The Team adopted a naming convention similar to that used for MER targets, where larger geologic features such as rocks or outcrops were named, while specific points or targets on those features contained the name of the parent feature as part of the target name. Acquired images were downloaded onto a laptop at Base Camp and analyzed by the Science Team, to determine whether changes in the original traverse were warranted. No changes were made.

#### Panorama

Eight images of the region were taken centered on Base Camp and spanning 360°. Images showed a massive structure of three stacked units over 100 m high, draped at the base by a talus concentrated in dark, light and mixed lobes. The sequence from bottom to top was determined to be: (1) lobes of dark and light talus, with some mixed areas, rising up to 60 m above Base Camp; (2) a layered buff-colored unit ~10 m thick; (3) a massive, blocky dark unit 40–50 m thick; (4) a dark capping unit of variable thickness. This stratigraphy is shown in Figure 5.

#### Station 1 (Enchilada)

A clast survey image looking downward from approximately 2 m high showed red, white, dark and light clasts overlying a buff unconsolidated material. Clasts appeared to be sub-mm and potentially rounded. Handlens-scale images of two clasts within this clast survey image (light-toned Tortilla and dark-toned Pinto Bean) revealed clasts with shapes and textures interpreted to be sedimentary and basaltic respectively. These were preliminarily tied to the light and dark units noted in the Panorama.

#### Station 2 (Chorizo)

A three-image mosaic of the 2-m tall boulder Chorizo at cm-scale revealed a mottled interior of dark clasts with lighter, orange exterior rims. In the single handlens-scale image acquired, vesicles could be resolved in the dark clasts. This boulder was interpreted to be spatter or agglutinated volcanic rock made of basaltic clasts with palagonitized rims. Figure 1b shows the image data acquired for this target.

#### Station 3 (mid-traverse)

**Figure 6:**
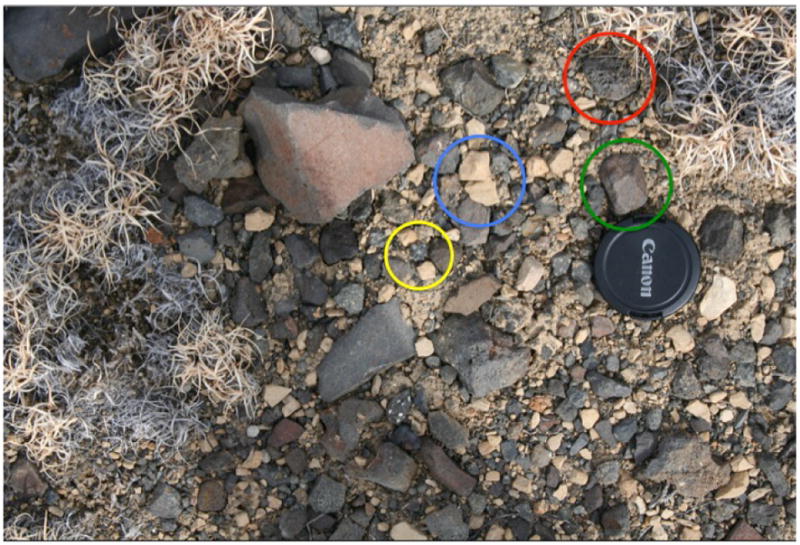
Mid-traverse clast survey, Station 3. This image typifies the diversity of clasts in the region, including cindery (red circle) and more massive (green circle) basalt, lighter-colored sedimentary fragments (blue circle), and dark, massive basalts with green or tan inclusions (yellow circle). Originally hypothesized to be amygdaloidal basalt, subsequent to observations of Station 6, this clast was interpreted to be a xenolith.

Following a three-image “drive-direction” mosaic and a single image upslope of Taco Bluff, the decision was made to take a clast survey image to document any changes in talus occurring upslope. This single image revealed a similar population of clasts as Station 1, with the exception of a few unusual mottled fragments (noted in Figure 6). These dark clasts were fine-grained, but contained discrete light or green grains with very distinct edges and sharp outlines. These were originally hypothesized to be amygdaloidal basalts. However, subsequent observations of clast litter and clasts at Station 6 led the Science Team to re-examine Station 3 data. Close examination of the clast survey image revealed that the green and tan inclusions were not confined to vesicles, suggesting that they were not amygdaloidal, but were instead genetically related to xenoliths.

#### Station 4 (Redchile and Greenchile)

**Figure 7:**
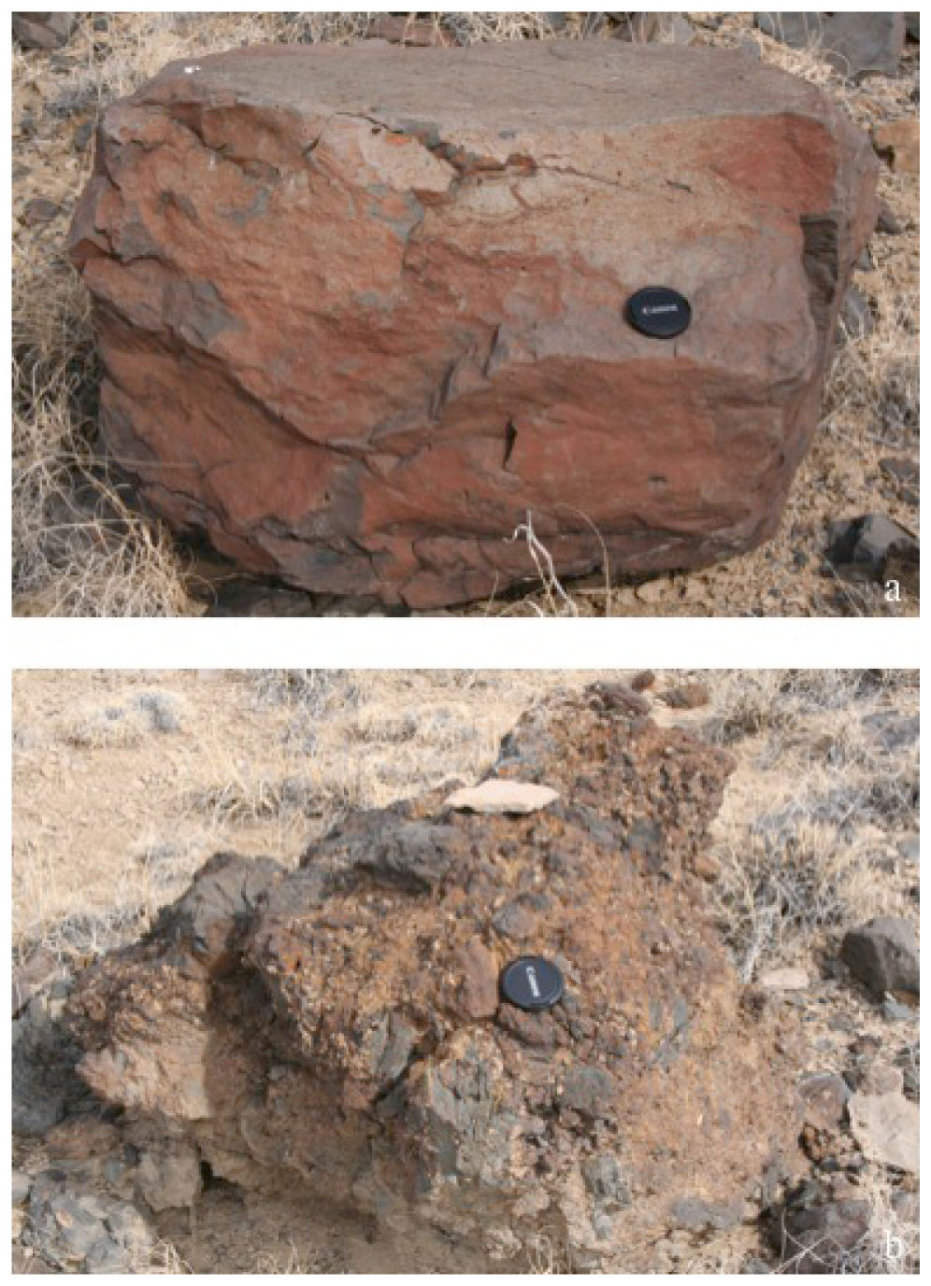
Targets from Station 4, Redchile (a) and Greenchile (b). These two targets represent the two primary basalt types of boulders seen in the region.

The Station 3 “drive-direction” mosaic revealed two m-scale boulders that were imaged at the cm-scale (three images each) and the μm-scale (one image each). These boulders are shown in Figure 7. Also imaged at cm-scale was Greenchile_Chip, a 7 cm diameter buff-colored clast found on top of the upslope boulder. The downslope boulder Redchile was a blocky, reddish vesicular boulder interpreted to be a basaltic boulder derived from either the dark upper unit or the dark capping unit. The upslope boulder Greenchile was observed to be a clastic, vesicular boulder similar in texture and morphology to Chorizo, and was thus interpreted to be spatter or an agglutinated volcanic rock with embedded, palagonitized clasts. Clasts embedded in Greenchile contained green-clear crystals interpreted to be olivine. Greenchile_Chip was a platy, buff-colored, granular clast composed of sub-angular to well-rounded sand-sized grains, and was interpreted to be a sandstone fragment derived from Taco Bluff.

#### Station 5

A three-image “drive-direction” mosaic allowed us to refine the location of Station 5. At this station (the outcrop Sopapilla), the Science Team acquired a single cm-scale clast survey image to document talus changes, and a 3-image mosaic of a portion of platy, light-toned outcrop. The morphology and texture of surface fragments all were similar to that of the outcrop, with no darker clasts present. This led to the interpretation that talus at this point in the slope was dominated by fragments of the Sopapilla outcrop. The outcrop images revealed light-toned, granular-textured rock covered with dark organics. Three μm-scale images were acquired, which resolved a matrix of sub-angular to rounded sub-mm clasts welded together. The outcrop itself showed no apparent layering, though it appeared to be weathering into platy fragments. The nature of the grain morphology and structure within the outcrop led the Science Team to interpret this outcrop as sandstone.

#### Station 6

**Figure 8:**
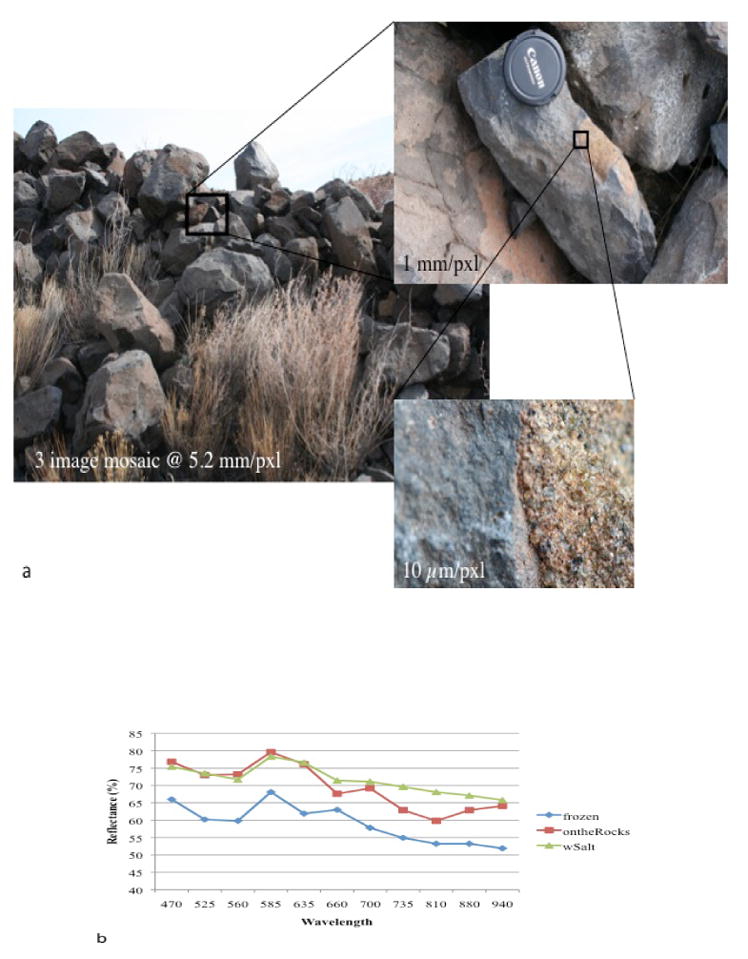
Targeted images and compositional data for station 6, Margarita Ridge: (a) Portion of ridge taken from a distance of 2 m, with ridge block taken from a distance of 0.5 m and xenolith in target Margarita_withsalt at 10 μm/pixel; (b) visible and infrared spectra of three targets from Margarita Ridge. Note the prominent absorption band around 1 μm for all targets, indicative of Fe-bearing phases.

The last station, referred to as Margarita Ridge, (Figure 8) was a long, relatively narrow ridge composed of loose, irregular, angular dark blocks. The ridge was imaged in a 3-image mosaic at a distance of 5 m, and then as a 3-image mosaic from a distance of 1 m. A single down-looking clast survey was also taken to document changes in talus. At this scale, dark, finely-vesiculated blocks and massive, fine-grained dark blocks were resolved, some with grey-white to orange inclusions up to 5 cm long axis. The Science Team was unable to estimate the number of rocks in this ridge that contained inclusions based on the images acquired.

Single images at μm-scale were also acquired of three separate targets, Margarita_withsalt, Margarita_ontherocks and Margarita_frozen. Margarita_withsalt was a massive-textured grey-to-white inclusion with a very narrow, discontinuous orange rim, while Margarita_ontherocks was an orange, granular-textured inclusion with green and clear crystals and a thicker orange rim. Margarita_frozen was a massive block with no inclusion, acquired as a basis for comparison. Taken together, the ridge was interpreted to be a rock fall of basaltic blocks from the dark upper unit, containing xenolithic inclusions of olivine and spinel with oxidized rims.

### Tiger Team observations

The Tiger Team took detailed observations of materials at each of the six stations, as well as several other stations. Observations from Science Team stations are enumerated in Table 1; observations at other stations are summarized here. The Tiger Team stations are noted in the order in which they were visited. Targets and stations also visited by the Science Team are noted in parentheses in this section. As noted earlier, the Tiger Team proceeded in a different order of traverse than the Science Team.

#### Station SC-10-1 (Stations 1 and 2, Enchilada and Chorizo)

The Tiger Team progressed directly to the boulder Chorizo (Station 2) and examined all sides. It was observed to be composed of welded reddish basaltic cinders partially palagonitized. After examining the exposure on the east side, the team recognized that it hosted abundant mantle xenoliths, many of them altered. The team then examined the float in the vicinity of Chorizo (this area partially overlapped with Science Team Station 2 – Enchilada). Sedimentary rocks and two dominant basaltic lithologies were identified: red, cindery basalt and darker, massive basalt (the latter appeared to be from a flow). Both basalt lithologies contained xenoliths, and they tended to be fresher in the massive basalt. The morphology and appearance of the boulder led the Tiger Team to hypothesize that it was derived from the reddish material on the mesa above the buff cliffs (Science Team dark unit).

#### Station SC-10-2 (Station 5, Sopapilla)

The Tiger Team proceeded next to SC-10-2 (Science Team Station 5), noting that the float along the way was similar to that seen in the vicinity of SC-10-1. Station SC-10-2 was a mound of layered, light-colored rock. It appeared to be sandstone but team members discussed the possibility that it was volcaniclastic in origin. The team hypothesized that the mound was a slump block from the buff cliffs (the Science Team’s buff unit), but noted that it appeared to be lighter (less reddish) than the cliffs and had a higher albedo on the photos than the cliffs, so the team cautiously rejected the hypothesis.

#### Station SC-10-3

The Tiger Team advanced to the cliffs of the buff unit, where the massive basalt float Chorizo was assumed to have come down. This station was a sandstone outcrop, similar to Station SC-10-2 but with fewer mafic minerals. The Tiger Team did not determine whether Stations SC-10-2 and SC-10-3 were variations of the same rock or represented different units.

#### Station SC-10-4

The Tiger Team traversed uphill along and to the base of the buff cliffs. These were determined to be another sandstone outcrop. The Tiger Team hypothesized that this station, along with stations SC10-2 and SC-10-3, are the same unit and the rock exposed above the talus are portions not yet eroded away.

#### Station SC-10-5

The Tiger Team then traversed up the valley/channel leading up to the base of the buff cliffs and the cliffs themselves. Examination of the cliffs revealed that the cliffs were primarily sandstone with a soft mineral identified using a fingernail scratch as gypsum (either primary or deposited by leaching and evaporation). Also present at the location along the base of the cliffs were massive blocks of basalt float containing xenoliths.

#### Retracing the Science Team Traverse

Once done with observations of Station SC-10-5, the Tiger Team traversed the Science Team stations in order, which involved revisiting some previously-examined stations. In some cases, the team took the opportunity to make more detailed observations of clast types and morphology, though these observations were not seen as a necessary component of the team’s traverse the first time through.

The Tiger Team re-examined Science Team stations 1–3, confirming previous observations and hypotheses. They then examined Station 4 (Redchile and Greenchile), two boulders that the team had not previously noted in their first traverse. The morphology and composition of these boulders led the team to connect them to the two types of basalt float they had observed in their traverse between stations SC-10-1 and SC-10-2. The team then reexamined SC-10-2 (Station 5, Sopapilla), confirming their previous observations, and moved to Science Team Station 6, Margarita Ridge, which extends along a transect between stations SC-10-4 and SC-10-5. Observations taken at this station and subsequently were of lherzolitic and clinopyroxenitic xenoliths embedded in basaltic blocks. The data from this station allowed the team to cement their hypothesis of the geologic history of the site. The Tiger Team hypothesized a cinder eruption of scoria, followed by flows of basalt onto the paleosurface (now the mesa top, the Science Team’s upper capping unit). Uplift and erosion formed the mesa and the massive basalt cascaded down the channel (probably moved by water and mass-wasting).

### Follow-on observations

On the third day of the field test, we had a debriefing among the Science and Tiger Team members, the results of which are summarized in the next section. Additionally, we acquired compositional data of samples taken by the Tiger Team and the “rover” by using the portable field spectrometer and XRD. Samples were not analyzed in situ, but were returned to the hotel to be analyzed, a scenario that does not precisely duplicate the MER situation in which targets are analyzed in situ by the Alpha Particle X-ray Spectrometer (APXS) or Mössbauer Spectrometer (MB) where time and data volume allow. This was a necessary step, however, in order to allow the fieldwork to be conducted within the short time available. Samples taken by the Science Team included Chorizo, Greenchile, Redchile, Greenchile_Chip, and the three targets from Margarita Ridge. Samples taken by the Tiger Team included weathered and non-weathered samples from Chorizo and Taco Bluff. Additionally, samples acquired by the Tiger Team from stations SC-10-1, SC-10-2 and SC-10-5 were analyzed by the Terra XRD.

Multispectral analysis of Science Team samples yielded results consistent with observations and interpretations of both the Science and Tiger Teams. Specifically, the spectral signature of a dark clast within Chorizo was relatively flat; no obvious spectral features were seen. However, the dark clasts within Greenchile, a target morphologically similar to Chorizo, showed a strong absorption band around 1.0 μm, consistent with Fe-bearing basalt. Redchile and Greenchile_Chip also displayed relatively featureless spectra, although the spectral signature of Greenchile_Chip increased in overall reflectance in the infrared. While not diagnostic, these results are not inconsistent with quartz-rich sand. For the three blocks sampled at Margarita Ridge, all displayed an absorption band around 1.0 μm that is consistent with Fe-bearing minerals common to basalt, as shown in Figure 8. Further, this absorption band is strongest for the two samples bearing inclusions, an expected result for olivine-bearing xenoliths. In short, the compositional data acquired did not require changes in hypotheses for either team.

Reliable XRD data were acquired for samples from Science Team stations 2 and 5 (the Tiger Team’s SC-10-1 and SC-10-2) and from the Tiger Team’s station SC-10-5. The sample from station SC-10-1 (Science Team station 2) is a smectite with calcite and plagioclase phases; this is fully consistent with the conclusion of both teams that it is a palagonitized basalt. The sample from SC-10-2 (near Science Team station 5) contains quartz/calcite with traces of dolomite, also consistent with both teams’ interpretation of provenance. The sample from station SC-10-5 is also a quartz/calcite/dolomite, with a trace of plagioclase. This data leads to the conclusion that the sample from SC-10-2 is indeed a slump block from the cliffs of SC-10-5, even though the Tiger Team rejected this hypothesis. The other minor discrepancy between the Tiger Team’s hypotheses and the XRD data was the identification of calcite. As certain facets of calcite can have similar hardness to gypsum, we believe the Tiger Team may have misidentified calcite as gypsum.

## Discussion

We compare the observations and interpretations of both teams, as listed in Table 1, in relation to how well each team was able to identify and characterize materials, and then use them to reconstruct the geologic history of the field site. We note two caveats in this discussion. Firstly, as noted earlier, though the Tiger Team visited the same stations as the Science Team (in addition to other stations), they did not examine them in the same order. Secondly, because of the nature of human observations, we must extrapolate from notes, rather than rigorously quantify, the number and type of observations the Tiger Team made, and then translate those observations into images acquired by a rover instrument.

Baseline data for the field site is derived from previous work (Crumpler et al. 1982, 2001, 2007; Hallett et al. 1992, 1997) and comprehensive field analysis by the local collaborator prior to the field test. Based on this standard, important materials that the teams could potentially have identified included sandstone, fresh and altered massive and basaltic scoria, basaltic ash, fine glass, and mantle xenoliths with diverse mineral compositions, derived from units shown in Figure 3.

### Assessment of the results of Science Team operational strategies

The operational strategies used by the Science Team provided adequate information for the Team to determine the general geology of the field site, which included a basic understanding of the stratigraphic sequence and identification of major units. Some regional context was also determined by examining “orbital” images in conjunction with ground-based observations. The Science Team identified several key geologic materials and characterized their morphology, although ash and glassy fragments were either not imaged, or were not imaged at a resolution high enough to identify them. The Science Team was able to identify the primary geologic processes that contributed to site history, and correctly identified the ones that the team sampled.

However, the data set acquired by the Science Team through MER rover-inspired operational strategies did not provide the information that would have led to a more nuanced understanding of the geologic history of the site. For example, there was insufficient time to acquire high-resolution follow-on data for the potential capping unit given the other priorities of the Science Team. Specifically, the similarity in morphology and appearance between the capping unit and the darker basalt unit led the Science Team to conclude that without sampling the capping unit directly, or imaging it at cm-scale (both highly time-intensive observational sequences), they would likely not be able to discriminate between talus from these two units; thus, determining the precise nature of the capping unit was given a lower priority than other observations. Therefore, the Science Team was unable to determine its nature, or whether any talus imaged in the clast surveys derived from this unit. Additionally, the Science Team nearly missed the presence of mantle xenoliths at the site, though they were ubiquitous and the Tiger Team observed them almost immediately. The nature of the discrete green and white “grains” within some of the dark clasts imaged at Station 3 was noted but not appreciated as indicating potential xenoliths, because the image was not analyzed in detail before moving on to take higher-priority images. Indeed, the Science Team members realized later in the day that they sat only 1 m away from a 7-cm diameter xenolith, but because the location was not on the traverse, they were compelled to ignore it. Thus, for this site, xenoliths were not broadly-distributed enough, or did not have a sufficiently unique expression at long-range resolutions for the Science Team to easily locate and identify them using MER-inspired operational strategies. We note that, at a field site with a more complex stratigraphy, such as the dry run on Day 1, the Science Team did not discover all major units present. This is consistent with our conclusion that the tested rover-inspired protocols were not sufficient to provide more than a first-order geologic picture of a field site.

Finally, it is important to note that the Science Team determined the location of stations and the priority in which they were studied very early on in the fieldwork, and these were never changed, though the Science Team did revisit the list of stations twice during the test. This, again, is a direct result of the rapid acquisition of data and the need to make science decisions just as rapidly. The Science Team did not have the time for in-depth debate of the traverse or station locations and had to rely on instinct for what appeared interesting from a distance.

### Assessment of the results of Tiger Team operational strategies

As noted above, the Tiger Team identified several geologic materials that the Science Team did not, including thin beds of fine-grained glass, ash, abundant cinders, and the presence of calcite in the lower sandstone layer. The Tiger Team also was able to use this additional information to hypothesize a history that included in order: sedimentary deposition and lithification, cinder cone eruptions, effusive basalt flows, and then uplift, excavation and downslope movement. Like the Science Team, the Tiger Team was ultimately unable to determine the emplacement history of the uppermost dark unit, or to confirm whether it was a capping unit. However, this was because the two members of the team could not agree on a hypothesis rather than because of a lack of information that would allow more than a rudimentary hypothesis to be posited. In short, the Tiger Team produced a much more detailed picture of the overall geologic history and stratigraphy of the site than the Science Team was able to produce. We believe this is due to three reasons: (1) the ability to manipulate samples to observe them at more favorable angles; (2) a more comprehensive approach to conducting observations, including the ability to observe and assimilate information during the traverse; and (3) more time available for observation and analysis rather than operational concerns. As the first point is not attributable to operational strategies, we discuss the latter two below.

In terms of the approach to observations, the traverse-mode operational strategy for MER, and thus the one utilized by the Science Team, has been one of reconnaissance (*e.g.*, Fong et al. (2008)). By contrast, while the investigations the Tiger Team chose to conduct were organic and not easily quantifiable, they may be described as comprehensive rather than representative. For example, the Science Team observations at Station 2 (the boulder Chorizo) consisted of a three-image mosaic taken at cm-scale from about 1 m distance, one cm-scale image taken from 0.3 m, and four μm-scale images comprising a mosaic. This data set constituted a reasonable approximation of both the unique and the representative features of this target. By contrast, the Tiger Team circumnavigated Chorizo several times, examined the boulder at numerous height levels, and studied several regions under a handlens before bagging a single sample. Indeed, it is at this target that the Tiger Team first discovered and examined in detail the mantle xenoliths at the site. This type of systematic examination is common for the field geologist but has been possible only rarely during the MER mission, and most often during the late stages, after the primary goals of the mission had essentially been met (e.g. the circumnavigation and three-dimensional mapping of the iron meteorite “Block Island” starting around sol 1961). The difference between taking representative and comprehensive observations of a target was seen here to equate to the difference between a first-level and a more detailed understanding of a site’s geologic history.

In terms of available time for observation and analysis, time “sinks” for the Science Team included: (1) the time required to conduct the operational activities needed to acquire data; and (2) the additional time needed to examine and analyze data as it was made available. Both of these will likely be issues in a continuous communications scenario on the Moon.

Operations tasks included choosing observations, “commanding” the camera, acquiring data, reading and analyzing that data, and then deciding based on the acquired data whether more observations or a change in the traverse or observational plans were warranted. One factor influencing the Tiger Team’s ability to successfully connect the in situ evidence to the regional geologic context was that it was not burdened with these tasks. For example, in the time it took the Science Team to acquire a three-image mosaic of Station 2 so that a handlens-scale target could be chosen, the Tiger Team had already executed their observations and had moved on to their next target. Interestingly, because of this lesser time constraint, and the consequent increase in available data, the Tiger Team had more opportunities to debate and pursue incorrect hypotheses, which the Science Team rarely did.

The Science Team also required significantly more time to analyze images as they became available, in order to come to similar conclusions as the Tiger Team. For example, while the Science Team was attempting to determine whether the lower buff layer was a sedimentary or volcanically-derived layer based on a handful of images, the Tiger Team had already determined the composition of this layer and was addressing a hypothesized stratigraphy inferred from samples which the Science Team had not acquired. A corollary to this point is that the Tiger Team had less need to prioritize each of its activities, and thus gathered the more tangential observations that were ultimately key to producing the more complex, nuanced history of the site.

Because of the considerations listed above, the goals of each team evolved along different paths. The hypotheses that the Science Team chose to address were more general, so their operational goals and tasks were different to match the requirements of these hypotheses. By contrast, the Tiger Team goals advanced and developed so rapidly with the addition of new information that they surpassed the original goals of the Science Team too quickly to record. Indeed, the Tiger Team settled on a model for the overall geologic picture so soon into the field test that bias may have crept in, in a way that did not occur with the Science Team.

## Lessons learned

The GHOST field experiment was designed to provide information regarding how, and how well, MER-based semi-autonomous rover operational strategies may maximize science return when used to conduct science in a lunar environment. Quantifying the results of this experiment in terms of “maximizing” science is not straightforward, however. Attempts have been made to quantify science return for remotely-conducted field geology, but many of these focus on the efficiency of the crew, robot or team in performing tasks (*e.g.*, Forrest et al. 2009; Bualat et al. 2010). Assigning numerical values to the process of scientific discovery remains problematic because it is, in fact, a process rather than a set of observations that, if met, will by definition produce a specified set of conclusions.

We can quantify observations related to rover instruments with relative ease, but while observations facilitate science, they are not science in and of themselves. Science is a heuristic process, depending on many mutually dependent qualitative factors, including the composition of the science team, the experience of each member (both scientifically and with the tactical process), and the geology of the field site itself. Because this test focuses so strongly on identifying, outlining and organizing our understanding of how science-driven operational strategies affect science return, we present our results as a list of lessons learned, rather than attempt to assign quantitative values to results that resist quantification.

(1) As is the case for MER, the Science Team spent a large amount of time conducting science operations, rather than analyzing science data. For example, the Science Team rarely had time to examine a full panorama before being required to make decisions regarding the traverse plan. As a consequence of this, some of the acquired data were never used to inform subsequent observations and analyses. Generalizing this result, the MER-inspired methodology is not conducive to utilizing all acquired data in a timely manner for the case of any lunar architecture that involves the acquisition of rover data in near real-time.

While one possible solution to this problem would be to disassociate scientists from the operations process, prior experience with the MER mission and the results of this study indicate that this is impractical and counter-productive. Science input is a required component of acquiring the most scientifically robust dataset, and conversely, understanding and participating in the tactical process of semi-autonomous rover data collection facilitates and enhances the science return. We also reject another possible solution, that of acquiring only the amount of data that can be immediately digested by a given science team. Even though understanding may be incomplete during mission operations, acquiring data produces legacy datasets from which future workers may gain a more nuanced understanding.

Instead, we recommend developing more effective ways to “triage” the data as it is made available. Ways to mitigate the time lost by conducting operations might include producing better tools for providing processed data more quickly to scientists in a near real-time situation, or on-board analysis tools that would automate certain target selection tasks (*e.g.*, Gulick et al. 2001; Pedersen et al. 2005; Castaño et al. 2007; Gilmore et al. 2008; Estlin et al. 2009; Woods et al. 2009). Such tools are currently being developed for MER. Another MER-derived idea, utilized at the 2010 Desert RATS (Research and Technology Studies) field test, is one of having both a long-term strategic science team and a day-to-day tactical science team (Yingst et al. 2011). In this strategy, the tactical science team conducts real-time operations and makes the immediate decisions regarding the science observations to be made. Data and tactical science team analyses are then passed to the strategic science team, which examines all available data and formulates long-term hypotheses. In practice, while the strategic team examined all available data, this caused significant problems, as there was no way to triage the important data. One adaptation of this system suggested by both D-RATS and this fieldwork is to triage targets during the tactical process, in order to point the strategic team to the highest priority data. Though this strategy was originally implemented within an architecture that was driven by human rather than semi-autonomous robotic fieldwork, this may be a workable solution that we will test in future studies.

(2) Related to the first point, when the Science Team was able to examine the data, it required more time to glean the same amount of information from a comparable image than it took the Tiger Team to process and understand what they observed using traditional field methods. For example, the Science Team noted unusually-colored clasts in the mid-drive clast survey acquired at Station 3, but did not realize the import of this observation until after it was too late to affect the tactical plan. Again, for a lunar mission with near real-time communications this factor will likely significantly lessen the potential science return.

The current operational strategy requires increased time for scientists to integrate data and make decisions, both in the short term for tactical decisions, and long-term, to formulate, test and analyze hypotheses, and it is not clear whether there is an “optimum” amount of time for this to occur. Some field sites are more straightforward than others, so there is no formula that may be applied to quantify the specific amount of time required for maximum science return. In a qualitative sense, the results of this experiment indicate that semi-autonomous rover missions should build in time for science to “happen”, outside the tactical process.

(3) For lunar missions where a primary goal is detailed analysis of stratigraphy and geologic processes, or for reconnaissance missions where the site is stratigraphically complex or subtle, a more comprehensive approach to conducting observations would yield better science return. The field experiment demonstrated explicitly the commonly accepted idea that geologists do not naturally follow the highly regulated protocols that are a required component of remote geology. For example, the Tiger Team deviated from the Science Team’s traverse almost immediately after getting to the field site; further, they chose to follow a different path to all stations. Thus, while the MER-derived operational strategies generally mimic those ideally followed by geologists in the field, the combination of heuristic knowledge gathering plus increased mobility mean that field geologists have much more flexibility, and use it to change traverses frequently. Conversely, however, the observations the Tiger Team made were more comprehensive and systematic, while those of the Science Team were more representative. An unexpected consequence of this is that the Science Team tended to be attracted to unique materials and exotic features, presumably in order to take representative observations of all possible units, features and materials. Systematic observations, such as “mid-drive” imaging and the clast surveys, were helpful in mitigating this issue somewhat, by revealing the variety of materials at the field site in a regularized way. Crucial contextual issues such as determining the nature of smaller or more distant units, however, were addressed catch-as-catch-can, or missed entirely.

In short, in a lunar environment with many different types of volcanic products, some of which are difficult to locate and identify, the MER-inspired methodology may be inadequate. To achieve a level of data acquisition that allows for detailed analysis of stratigraphy and geologic processes, an operational strategy should require a planned set of systematic observations, and exotics that cannot be put into context should be considered less valuable in a scientific sense and thus a lower priority for analysis. To achieve such a mission objective for future lunar missions that have similar instruments as a MER-type rover, it is likely that these systematic observations will need to be acquired even at the cost of data volume and mission time.

For a mission with the goal of initial reconnaissance (such as MER), the traverse-mode operational strategy derived from the MER mission is appropriate for use on the Moon, given the recommendations made above. But if the goals of the lunar mission are different — for example, identification and characterization of a specific resource, such as ice at polar regions — the science strategies will likely need to be altered. The current strategies tend to support representative observations, while a mission with the goal of finding a specific material, or a specific sample type, would require a much more systematic set of observations. Such a strategy would require significant additional engineering and computational resources, as it is precisely the limited time, data volume and wear-and-tear on rover instruments and other components that prevented the MER rovers from adopting a strategy that encompassed more systematic observations. A reconnaissance pass using MER-derived observational strategies followed by systematic work with a different sequence of observations adapted to a more comprehensive search is one possible solution. Other solutions might involve increasing the number of available rovers rather than simply increasing the number of observations a single rover could take. Another possibility would include increased mobility, perhaps at the expense of instrumentation. Such solutions, however, would require that the current rover strategies be significantly altered to adapt to multiple rovers or repeated traverses, and at this time it is unclear whether a multiple-rover architecture is a likely one for lunar exploration. In the short term, we will conduct fieldwork to test how MER-inspired single-rover strategies may be adapted to more systematic observations by conducting a similar field experiment with the primary goal of identifying and characterizing a specific resource, such as water ice.

Finally, it is important to note how all of these issues are inter-related. Time-limitation is a well-known constraint of any planetary mission situation, whether or not it involves humans on the surface. Remote semi-autonomous rover fieldwork utilizes non-renewable, expendable resources embodied in the rover and its instruments, and these must be used judiciously in order for science priorities to be met before resources are spent. It is also true that any terrestrial field experiment is time-limited; geologists must plan field experiments within limited timeframes. However, one point that became evident during the GHOST field experiment was that, regardless of how time-limited a terrestrial field experiment may be, that limitation is of a different sort than that experienced during a planetary mission. The terrestrial field geologist is aware that, no matter the location of the field site, additional research on that site almost always exists in the literature, and a return trip is always a possibility. This is not the case in a planetary situation, and thus the perception of limited time is much more explicit. Added to this perception is the fact that semi-autonomous rover operations and data analysis require more time to execute than traditional field methods. We believe that this combination of pressures on the time of scientists plays an important part in driving the strategy of representational rather than comprehensive observations.

## Figures and Tables

**Table 1:** Observations and interpretations of Science and Tiger Teams at Cerro de Santa Clara.

	Science Team			Tiger Team		
Station	Data Collected	Observations	Interpretations	Data Collected	Observations	Interpretations
None	Orbital imagery 360° panorama of 10 images	Talus from outcrop slopes up to outcrop. Thick, darker unit lies above a thinner buff unit. Dark, thin unit caps the stack.	Original interpretation at this scale is a basaltic unit underlain by a sedimentary unit.	Visual scan of the region (equivalent of 360° panorama).	Mesa of dark and buff outcrop, surrounded by talus.	Basaltic dark unit, overlying buff-colored sedimentary unit. Talus and float derived from overlying dark and buff units.
1. Enchilada	1 cm-scale image looking down Handlens (μm)-scale color images of two targets, Tortilla (light) and Pinto bean (dark)	Red, white, dark and light clasts litter the surface, in a buff matrix. Clasts appear to be clastic, sub-mm and potentially rounded. Target Tortilla is buff-colored, sub-round and granular-textured, with coarse grains. Target Pinto bean is dark, massive, and angular, with a matrix of just-resolvable grains.	Dark clasts derive from dark unit; light clasts derive from light unit. Dark clasts may be volcanic; light clasts may be sedimentary.	Visual inspection of surface particles; cm-scale examination, including picking up numerous samples; two representative samples chosen for later compositional analysis.	Surface litter composed of the following: Loose particles of light and dark basalt, including basalt clasts (vesicular are rounded, massive are angular), 1–20 cm diameter. Basalt particles mixed with some vesicular reddish, rounder particles (some xenoliths visible, 2–3 cm across). Buff to orange-red clasts. Buff-colored fine-grained regolith.	Dark clasts derive from dark unit; light clasts derive from light unit.
2. Chorizo, large boulder	3-image mosaic at cm-scale. Single cm-scale image taken from 0.3 m distance. Four-image handlens (μm)-scale color mosaic of basalt clast target, Chorizo_pork.	Large (2x3 m) mottled boulder, visible from airphotos; orange and black domains visible in m-scale images. Dark, vesicular clasts with lighter, orange exterior rims	Spatter or agglutinated volcanic rock. Clasts with palagonitized rims.	Tiger Team Station 1 Visual inspection around circumference of boulder; cm-scale examination of several targets; one sample taken.	Boulder is 2x3x5 m, generally basaltic, locally very finely vesiculated, with varying lithic clasts. Vesicles not uniformly distributed. Orange, with rounded grains, possible zeolites in vugs. Some orange clasts w/distinct borders, up to 4 cm diameter Largest basalt clasts are 30–40 cm. Some litter around the boulder contains xenoliths up to 4 cm diameter, of orange and green crystals, with quench textures.	Nodule-rich boulder with secondary grains indicating that the boulder is large cinders/clasts welded together. Boulder source is red layer above buff cliffs, 80–95 m vertically higher. Litter is pyroxene or olivine-rich xenoliths in basaltic clasts.
3. Mid-traverse at N40°E, imaging of Taco Bluff	Drive direction 3-image mosaic from target Chorizo. 1 cm-scale image looking down	Red, white, dark and light clasts on a buff soil. Most clasts appear to be clastic, sub-mm and potentially rounded. Three dark clasts contain discrete light or green grains or crystals in a fine-grained matrix; edges very distinct and outlines are sharp.	Talus fragments similar to those found at Station 1. Unusual clasts with embedded grains or crystals may represent xenoliths.	Visual inspection of loose particles along traverse from Station 2 to Station 4; cm-scale examination, including picking up numerous samples.	Litter consists of grayish basalt cobbles up to 40 cm diameter, some displaying surface spheroidal weathering. Less vesicular clasts than Station 1. Xenoliths visible; weathering red, fresh are green.	Float diversity is similar across the site, including red, cindery basalt and darker, massive basalt; and sedimentary rocks. Float is derived from outcrop.
4. Redchile and Greenchile	3-image mosaic at cm-scale of each boulder. Single cm-scale image taken from 0.3 m distance. Three handlens (μm)-scale color images of Redchile; five μm-scale color images of Greenchile”; one μm-scale image of fragment Greenchile_Chip on Greenchile.	Redchile is a blocky, reddish vesicular boulder. Greenchile is a clastic, vesicular boulder similar to Chorizo in texture and morphology. Embedded clasts contain green-clear crystals. Chip is a platy, light-toned, granular clast composed of sub-rounded to well-rounded sand-sized grains.	Redchile is a basaltic boulder derived either from the dark unit or the dark capping unit. Greenchile is spatter or agglutinated volcanic rock, containing clasts with palagonitized rims. Chip is interpreted to be a sandstone derived from “Taco Bluff”.	Visual inspection around circumference of boulders; cm-scale examination of several targets.	Two boulders visible. Redchile is a fine-grained boulder with a red-grey exterior. Greenchile is a vesicular, cindery boulder with some visible xenoliths	Greenchile is a clinker breccia associated with Station 5, Sopapilla. Redchile is a basaltic boulder. Both boulders are connected to the two types of basaltic float seen throughout the site.
5. Sopapilla	1 cm-scale image looking down 3-image mosaic at cm-scale 3-image mosaic at handlens (μm)-scale in color	Surface clasts are all light-toned; dark-toned basalts and other clasts are absent. Some surface clasts contain darker angular fragments. Light-toned, granular-textured outcrop with dark organics. μm-scale images show sub-angular to very angular sub-mm clasts apparently welded. No apparent layering, but outcrop is weathering out in platy, blocky chunks.	Outcrop appears to be welded angular volcanics.	Tiger Team Station 2 Visual inspection around circumference of boulder; cm-scale examination of several targets; two representative samples taken.	Mound of platy, buff-colored sandstone, containing quartz, feldspar and local green-colored mineral. Grains are ≪1–2 mm diameter, sub-round and well-sorted. No outcrops are in the mound, but there is a stratigraphic boundary between the red lobate basalts and the sediments.	Soft, submature sediments, varying in size, a range of minerals (black, red, quartz). Interpreted to be a slump block from lighter-colored layers just uphill.
6. Margarita Ridge	3-image mosaic at a distance of 5 m. 3-image mosaic at a distance of 1 m. 1 cm-scale image looking down μm-scale images of three blocks	Irregular, angular, dark-colored blocks concentrated in a ridge. Blocks include dark, finely-vesiculated blocks and massive, fine-grained dark blocks, some with inclusions up to 5 cm long axis. Inclusions are light-colored, granular, with green and clear crystals visible. Orange rims surround most inclusions.	Angular blocks are interpreted to be a rock fall. Inclusions are interpreted to be xenoliths containing olivine and spinel, with orange alteration rims.	Tiger Team Station 4 Visual inspection; cm-scale examination of several targets; representative samples taken.	Basalt/xenolith tongue lies on buff colored cliff at an altitude higher than the Station 2 mound. Sand grains, immature, round to sub-round with mafics. Buff rocks are visible all the way up the valley cut.	Lava ridge is landslide from the table/cap basalt above; these armor the soft rock. Buff rocks continue as one unit
